# Supplementary figures and images for: De novo assembly of Iron-Heart Cunninghamia lanceolata transcriptome and EST-SSR marker development for genetic diversity analysis
Source: PLoS One. 2023 Nov 2;18(11):e0293245. doi: 10.1371/journal.pone.0293245 (PMC10621985; doi:10.1371/journal.pone.0293245)

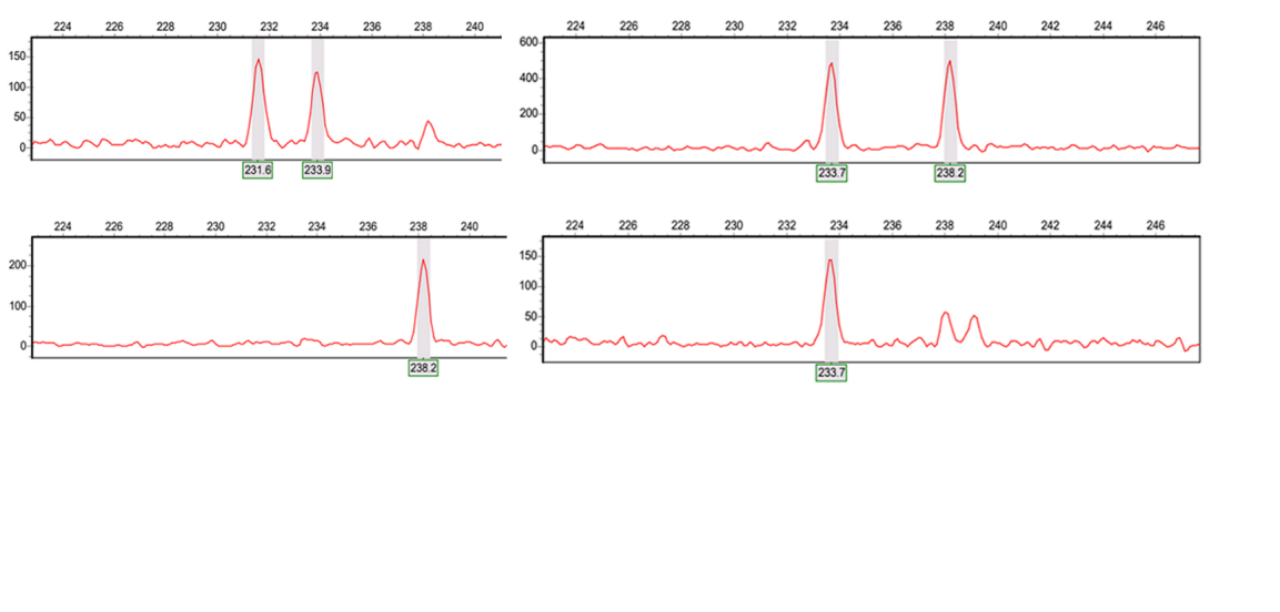

Supplement: S1 Fig — (TIF) [file pone.0293245.s004.tif]
