# Supplementary material for: De novo assembly of Iron-Heart Cunninghamia lanceolata transcriptome and EST-SSR marker development for genetic diversity analysis
Source: PLoS One. 2023 Nov 2;18(11):e0293245. doi: 10.1371/journal.pone.0293245 (PMC10621985; doi:10.1371/journal.pone.0293245)
Supplement: Attachment — Submitted filename: PONE-D-22-35149_review_06.03.2023.pdf [file pone.0293245.s007.pdf]

PLOS ONE

De novo assembly of Iron-Heart Cunninghamia lanceolat a transcriptome using Illumina sequencing and EST-SSR marker development for genetic diversity analysis of Chinese fir

--Manuscript Draft--

**Manuscript Number:**	PONE-D-22-35149
**Article Type:**	Research Article
**Full Title:**	De novo assembly of Iron-Heart Cunninghamia lanceolat a transcriptome using Illumina sequencing and EST-SSR marker development for genetic diversity analysis of Chinese fir
**Short Title:**	De novo assembly of Iron-Heart Cunninghamia lanceolata transcriptome using Illumina sequencing

**Corresponding Author:**	Can Xiao<br>Central South University of Forestry and Technology<br>CHINA

**Keywords:**	Iron-Heart C. lanceolata; Transcriptome; Function annotation; EST-SSR

Abstract:

As a wild relative of Chinese fir, Iron-Heart Cunninghamia lanceolata has a ~~large~~ heartwood ratio and excellent wood properties. ~~The gene pool contains excellent timber forests, and the species has important genetic and breeding value.~~ However, genetic studies on Iron-Heart C. lanceolata have been hindered by the limited genomic resources and markers. In this study, we sequenced the transcriptome of Iron-Heart C. lanceolata leaves using Illumina NovaSeq 6000, and we assembled and analyzed it using Trinity and MISA software. We used Primer 6.0 to design the SSR markers. We summarize the results as follows: (1) We obtained a total of 45,326,576 clean reads and 15,501 unigenes via transcriptome sequencing, splicing, assembly, and de-redundancy. We compared the unigenes in five functional databases. A total of 26,278 unigenes were successfully annotated in at least one database, and a total of 6,693 unigenes were annotated in all five databases, accounting for 5.79% of the total. The UniProt and Pfam databases contained annotations of 22,673 and 18,315 unigenes, respectively. In the GO database, the 23,963 successfully annotated unigenes were placed into three categories. In the KEGG database alignment, 10,196 unigenes were successfully annotated, which were placed into five categories: metabolism, which was the largest; genetic information; biological systems; cellular processes; and environmental information processing. (2) We obtained a total of 17,362 SSRs from all the unigenes, with a distribution frequency of 4.88%, of which mononucleotides were the main repetition type, accounting for 64.6%, followed by trinucleotides, accounting for 20.2% dinucleotides, accounting for 12.9%, and four, five, and six nucleotides, accounting for 2.3% of the total. The primary three-base ~~repeats~~ were AAG/TTC and GAA/CTT, accounting for 1.81% and 1.84% of the total, respectively, and the primary two-base repeat was TA/AT, accounting for 5.29% of the total, followed by AG/TC and CT/GA, accou[illegible]g for 2.50% and 2.53% of the total, respectively. In addition, the numbers of ~~repeats~~ of the different repeating bases in the SSRs substantially varied between 5 and 84, ~~and they were mainly concentrated from 5 to 23. The overall trend was as follows: the number of repeats decreased as the number of repeats increased, and the number of repeats decreased as the number of repeat bases increased.~~ (3) In order to verify the versatility and polymorp[illegible]n of the SSRs, we randomly selected 200 pairs of primers to amplify the Iron-Heart [illegible]nceolate ~~samples~~, and we screened a total of 15 pairs of polymorphic SSR primers. We performed the genetic relationship identification of Chinese fir plants of six different origins with the 15 SSRs, and according to the clustering results, the 15 pairs of SSR primers could effectively distinguish the samples of different origins. The present study provides a ~~theoretical basis~~ for the development of molecular markers of Iron-Heart C. lanceolata, ~~the improvement in and cultivation of new varieties, analyses of the genetic diversity,~~ and molecular-marker-assisted breeding.

Order of Authors:

sen liu

Gongxiu He

Powered by Editorial Manager® and ProduXion Manager® from Aries Systems Corporation

	Gongliang Xie
	Yamei Gong
	Ninghua Zhu
	Can Xiao
**Additional Information:**	
**Question**	**Response**
**Financial Disclosure**<br><br>Enter a financial disclosure statement that describes the sources of funding for the work included in this submission. Review the submission guidelines for detailed requirements. View published research articles from *PLOS ONE* for specific examples.<br><br>This statement is required for submission and **will appear in the published article** if the submission is accepted. Please make sure it is accurate.<br><br>**Unfunded studies**<br>Enter: *The author(s) received no specific funding for this work.*<br><br>**Funded studies**<br>Enter a statement with the following details:<br>• Initials of the authors who received each award<br>• Grant numbers awarded to each author<br>• The full name of each funder<br>• URL of each funder website<br>• Did the sponsors or funders play any role in the study design, data collection and analysis, decision to publish, or preparation of the manuscript?<br>• **NO** - Include this sentence at the end of your statement: *The funders had no role in study design, data collection and analysis, decision to publish, or preparation of the manuscript.*<br>• **YES** - Specify the role(s) played.<br><br>* typeset	The author(s) received no specific funding for this work.
**Competing Interests**<br><br>Use the instructions below to enter a competing interest statement for this submission. On behalf of all authors, disclose any competing interests that	NO authors have competing interests

could be perceived to bias this work—acknowledging all financial support and any other relevant financial or non-financial competing interests.<br><br>This statement is required for submission and **will appear in the published article** if the submission is accepted. Please make sure it is accurate and that any funding sources listed in your Funding Information later in the submission form are also declared in your Financial Disclosure statement.<br><br>View published research articles from *PLOS ONE* for specific examples.<br><br>**NO authors have competing interests**<br><br>Enter: *The authors have declared that no competing interests exist.*<br><br>**Authors with competing interests**<br><br>Enter competing interest details beginning with this statement:<br><br>*I have read the journal's policy and the authors of this manuscript have the following competing interests: [insert competing interests here]*<br><br>* typeset	
**Ethics Statement**<br><br>Enter an ethics statement for this submission. This statement is required if the study involved:<br><br>• Human participants<br>• Human specimens or tissue<br>• Vertebrate animals or cephalopods<br>• Vertebrate embryos or tissues<br>• Field research<br><br>Write "N/A" if the submission does not require an ethics statement.	N/A

General guidance is provided below. Consult the submission guidelines for detailed instructions. **Make sure that all information entered here is included in the Methods section of the manuscript.**<br><br>**Format for specific study types**<br><br>**Human Subject Research (involving human participants and/or tissue)**<br>• Give the name of the institutional review board or ethics committee that approved the study<br>• Include the approval number and/or a statement indicating approval of this research<br>• Indicate the form of consent obtained (written/oral) or the reason that consent was not obtained (e.g. the data were analyzed anonymously)<br><br>**Animal Research (involving vertebrate animals, embryos or tissues)**<br>• Provide the name of the Institutional Animal Care and Use Committee (IACUC) or other relevant ethics board that reviewed the study protocol, and indicate whether they approved this research or granted a formal waiver of ethical approval<br>• Include an approval number if one was obtained<br>• If the study involved *non-human primates*, add *additional details* about animal welfare and steps taken to ameliorate suffering<br>• If anesthesia, euthanasia, or any kind of animal sacrifice is part of the study, include briefly which substances and/or methods were applied<br><br>**Field Research**<br><br>Include the following details if this study involves the collection of plant, animal, or other materials from a natural setting:<br>• Field permit number<br>• Name of the institution or relevant body that granted permission	
**Data Availability**<br><br>Authors are required to make all data	Yes - all data are fully available without restriction

Powered by Editorial Manager® and ProduXion Manager® from Aries Systems Corporation

underlying the findings described fully available, without restriction, and from the time of publication. PLOS allows rare exceptions to address legal and ethical concerns. See the PLOS Data Policy and FAQ for detailed information.<br><br>A Data Availability Statement describing where the data can be found is required at submission. Your answers to this question constitute the Data Availability Statement and **will be published in the article**, if accepted.<br><br>**Important:** Stating 'data available on request from the author' is not sufficient. If your data are only available upon request, select 'No' for the first question and explain your exceptional situation in the text box.<br><br>Do the authors confirm that all data underlying the findings described in their manuscript are fully available without restriction?	
**Describe where the data may be found in full sentences. If you are copying our sample text, replace any instances of XXX with the appropriate details.**<br><br>• If the data are **held or will be held in a public repository**, include URLs, accession numbers or DOIs. If this information will only be available after acceptance, indicate this by ticking the box below. For example: *All XXX files are available from the XXX database (accession number(s) XXX, XXX.*).<br>• If the data are all contained **within the manuscript and/or Supporting Information files**, enter the following: *All relevant data are within the manuscript and its Supporting Information files.*<br>• If neither of these applies but you are able to provide **details of access elsewhere**, with or without limitations, please do so. For example:<br><br>*Data cannot be shared publicly because of [XXX]. Data are available from the*	All relevant data are within the manuscript and its Supporting Information files.

XXX Institutional Data Access / Ethics Committee (contact via XXX) for researchers who meet the criteria for access to confidential data.<br><br>*The data underlying the results presented in the study are available from (include the name of the third party and contact information or URL).*<br>• This text is appropriate if the data are owned by a third party and authors do not have permission to share the data.<br><br>* typeset	
Additional data availability information:	Tick here if the URLs/accession numbers/DOIs will be available only after acceptance of the manuscript for publication so that we can ensure their inclusion before publication.

Manuscript Click here to access/download;Manuscript;Manuscript.pdf

De novo assembly of Iron-Heart *Cunninghamia lanceolata* transcriptome using Illumina sequencing and EST-SSR marker development for genetic diversity analysis of Chinese fir

Sen Liu[1], Gongxiu He[1], Gongliang Xie[1], Yamei Gong[1], Ninghua Zhu[1,3], Can Xiao[2,*]

1.Faculty of Forestry, Central South University of forestry and Technology, Changsha 410004, China;

2.Jiangxi environmental engineering vocational college, Ganzhou 341000, China;

3.National long-term scientific research base for forestry in mid-subtropics China, Central South University of forestry and Technology, Changsha 410004, China

* Correspondence:17916370@qq.com

Abstract: As a wild relative of Chinese fir, Iron-Heart *Cunninghamia lanceolata* has a large heartwood ratio and excellent wood properties. The gene pool contains excellent timber forests, and the species has important genetic and breeding value. Wild biological germplasm resources have substantial application potential in the biological industry, and they are inseparable from the protection of global biodiversity. However, genetic studies on Iron-Heart *C. lanceolata* have been hindered by the limited genomic resources and markers. In this study, we sequenced the transcriptome of Iron-Heart *C. lanceolata* leaves using Illumina NovaSeq 6000, and we assembled and analyzed it using Trinity and MISA software. We used Primer 6.0 to design the SSR markers. We summarize the results as follows: (1) We obtained a total of 45,326,576 clean reads and 15,501 unigenes via transcriptome sequencing, splicing, assembly, and de-redundancy. We compared the unigenes in five functional databases. A total of 26,278 unigenes were successfully annotated in at least one database, and a total of 6,693 unigenes were annotated in all five databases, accounting for 5.79% of the total. The UniProt and Pfam databases contained annotations of 22,673 and 18,315 unigenes, respectively. In the GO database, the 23,963 successfully annotated unigenes were placed into three categories. In the KEGG database alignment, 10,196 unigenes were successfully annotated, which were placed into five categories: metabolism, which was the largest; genetic information; biological systems; cellular processes; and environmental information processing. (2) We obtained a total of 17,362 SSRs from all the unigenes, with a distribution frequency of 4.88%, of which mononucleotides were the main repetition type, accounting for 64.6%, followed by trinucleotides, accounting for 20.2% dinucleotides, accounting for 12.9%, and four, five, and six nucleotides, accounting for 2.3% of

the total. The primary three-base repeats were AAG/TTC and GAA/CTT, accounting for 1.81% and 1.84% of the total, respectively, and the primary two-base repeat was TA/AT, accounting for 5.29% of the total, followed by AG/TC and CT/GA, accounting for 2.50% and 2.53% of the total, respectively. In addition, the numbers of repeats of the different repeating bases in the SSRs substantially varied between 5 and 84, and they were mainly concentrated from 5 to 23. The overall trend was as follows: the number of repeats decreased as the number of repeats increased, and the number of repeats decreased as the number of repeat bases increased. The length of the SSR marker sequences was between 10 and 84 bp, of which 96.16% of the SSR fragments were between 10 and 29 bp in length, and only 3.84% of the markers were greater than or equal to 30 bp in length. (3) In order to verify the versatility and polymorphism of the SSRs, we randomly selected 200 pairs of primers to amplify the Iron-Heart *C. lanceolate* samples, and we screened a total of 15 pairs of polymorphic SSR primers. We performed the genetic relationship identification of Chinese fir plants of six different origins with the 15 SSRs, and according to the clustering results, the 15 pairs of SSR primers could effectively distinguish the samples of different origins. The present study provides a theoretical basis for the development of molecular markers of Iron-Heart *C. lanceolata*, the improvement in and cultivation of new varieties, analyses of the genetic diversity, and molecular-marker-assisted breeding.

Keywords: Iron-Heart *C. lanceolata*; Transcriptome; Function annotation; EST-SSR

Introduction

Iron-Heart *C. lanceolata* is an endemic tree species in Hunan and a relative of Chinese fir. It has a narrow habitat and relatively small number of individuals. Iron-Heart *C. lanceolata* is a wild plant with a small population that is only distributed in the Xiaoxi National Nature Reserve in Hunan province [1]. Its heartwood ratio is large, and the wood is hard, dark brown, shiny, and resistant to rot. Its wood properties are better than those of fast-growing Chinese fir, red-heart Chinese fir, and common fir, and it is the most ideal gene bank for commercial timber forests [2]. Due to its late discovery ~~and backward research~~, the current research on Iron-Heart *C. lanceolata* is in the budding and exploratory stage, ~~but there are only a few relevant~~ studies on the genetic structure of the population [3], seed quality [4], wood properties [2], ~~genetic transcriptome, molecular markers,~~

Restricted in wild. Discuss about its threatened potential.

~~etc.~~ As the international market demand has changed from the original emphasis on the quantity of the wood to an equal emphasis on the quantity and quality of it, the quantity and quality of the existing Chinese fir varieties are not sufficient to meet future production needs. Therefore, studies on Iron-Heart *C. lanceolata* are essential. At present, Iron-Heart *C. lanceolata* is relatively intact and not too affected by human interference; however, its traffic is blocked, which greatly limits its protection, utilization, and development.

Please improve the clarity ("the traffic is blocked").

~~Simple sequence repeats (SSRs), which are also known as microsatellites,~~ are short repeating DNA motifs (1–6 bp) that are arranged in tandem and that may vary in the number of repeats at a given locus [5,6]. Due to their advantages of high polymorphism, strong stability, good repeatability, easy detection, simple operation, and low cost, researchers have widely used them in molecular-assisted breeding research, such as on parental identification [7,8], population genetic structures [9,10], fingerprint construction [11,12,13], and association analyses of important traits [14,15]. ~~According to the source, we can divide~~ SSR markers ~~into~~ whole-genome ~~sequences~~ (g-SSR) and expressed sequence tag (EST-SSR) ~~markers~~ [16-18]. ~~G-SSR markers are developed based on genomes, which are costly and cumbersome, while EST-SSR markers are developed based on transcriptome-expressed sequence tags.~~ EST-SSR markers are easier to develop for plants with ~~incomplete genome sequences~~ and large genomes [19-21]. Although the polymorphism of EST-SSR markers is lower than that of g-SSR markers, it has better generality and transferability, a shorter development time, and it is directly related to the gene function, ~~and especially within the same genus~~ [16,17,22,23]. ~~In recent years, with the development of plant genome and functional genome research, large-scale plant gene sequencing has generated a large number of EST sequences, which researchers have uploaded to the public nucleic acid databases. The number is still increasing, and EST sequences have become the key resource in the development of EST-SSR [24-27]. However, with the rapid development of high-throughput sequencing technology, RNA sequencing provides a new method for studying transcriptome information [28,29]. We can not only use this technology to directly obtain a large amount of EST information, but also for SSR molecular marker development.~~ ~~At present~~, researchers have ~~carried out the development of~~ EST-SSR markers based on transcriptome data in many model and nonmodel plants [30,31,21], and especially in conifers, such as Korean pine [32], Masson pine [33], Chinese fir [34], etc. According to the above research ~~results~~, the use of plant transcriptome sequencing to obtain SSR markers ~~is~~

feasible and convenient. Although some markers were developed based on genomes or transcriptomes in Chinese fir, we found that the polymorphism and number of markers could not meet the experimental requirements during our research, and we could not analyze the fine spatial genetic structure of Iron-Heart *C. lanceolata* [35]. We need to develop ~~SSR markers~~ to accelerate the marker-assisted breeding of Iron-Heart *C. lanceolata.*

In the present study, our main objectives were as follows: (1) the development of reliable SSR primers ~~based on the transcriptome data and analysis of the SSR distribution~~ in Iron-Heart *C. lanceolata*; (2) the ~~selection of polymorphic, specific, and stable SSR markers, and the use of these markers~~ to identify the genetic relationship among Chinese fir varieties of six different origins; (3) the validation of the reliability of the SSR markers based on the transcriptome of Iron-Heart *C. lanceolata.* This study provides a reference for the ex situ conservation, fine spatial genetic structure analyses, and molecular-assisted breeding of Iron-Heart *C. lanceolata* at the genome level.

redundant from previous point

Materials and Methods

Plant Materials

We collected the seeds from the Hunan Xiaoxi National Nature Reserve in 2019, which were sown in the Botanical Garden of Central South University of Forestry and Technology in March 2020, and we collected the leaves of the Iron-Heart *C. lanceolata* seedlings in December 2020, which we wrapped in tin foil and ~~quick-froze with~~ liquid nitrogen. Igeneb[illegible] (Wuhan, China) completed the transcriptome sequencing. We obtained the materials used for the SSR primer selection and application from Chinese fir plants of six different origins collected in the Chaling Chinese fir germplasm resource collection nursery in Hunan. We collected 5–6 plants from each origin. We present the detailed information and codes of the samples in Table 1.

Table 1. Information on Chinese fir plants of six different origins.

Origin	Species	Altitude	Location	MAP (mm)	Code
Hunan, Yongshun	Iron-Heart *C. lanceolata*	849 m	E 110°15′, N 28°48′	1357 mm	HN-YS

Fujian, Shunchang	*C. lanceolata*-020	1295 m	E 117°45′, N 27°10′	1688 mm	FJ-020
Fujian, Shunchang	*C. lanceolata*-061	1383 m	E 117°45′, N 27°10′	1688 mm	FJ-061
GuangXi, Chenshan	Red-heart Chinese fir	1135 m	E 114°35′, N 27°20′	1663 mm	GX-CS
Hunan, Zhangjiajie	Fast-growing Chinese fir	265 m	E 110°40′, N 29°20′	1973 mm	HN-ZJJ
Hunan, Youxian	Chinese fir	143 m	E 27°01′, N 113°15′	1410 mm	HN-YX

Sequencing and annotation

We quickly ground the leaves of Iron-Heart *C. lanceolata* in liquid nitrogen until powdered, extracted the RNA, evaluated the purity and integrity, and then constructed a sequencing library. ~~We performed and filtered the high-throughput s~~equencing using the Illumina NovaSeq 6000 platform. We used Fast QC to evaluate the quality of the original reads, quickly remove reads with adapters, N reads greater than 10%, and low-quality reads, and we finally obtained the clean reads. We used Trinity software [36] to splice the reads to obtain the transcript fragments, and we used Corset [37] to perform the hierarchical clustering to obtain the nonredundant unigenes. We aligned and annotated the assembled sequences in the following five databases: Protein Families (Pfam) (http://pfam.sanger.ac.uk/); Universal Protein (UniProt) (https://www.uniprot.org/); Gene Ontology (GO) (http://www.geneontology.org); Kyoto Encyclopedia of Genes and Genomes (KEGG) (https://www.genome.jp/kegg/); Pathway (https://www.genome.jp/kegg/pathway/).

Should be detailed.

How, which software?

SSR design

We used MISA (http://pgrc.ipk-gatersleben.de/misa/) software to search the SSR markers in the unigene sequences, and the length of each repeat sequence was greater than or equal to 18 bp. After identifying the SSR marker, we used Primer 6.0 software (Premier Biosoft International, Palo Alto, CA, USA) to design the primers. The primer design adhered to the following principles:

Did you use the full unigene set to search for SSRs?

primer length: 18–25 bp; Tm value: 52.0°C–60.0°C; Tm value of upstream and downstream primers: within 5°C; (G + C) content: 40%–60%; primer amplification length: 100 bp–300 bp.

EST-SSR Screening and Relationship Identification of Chinese fir

We randomly selected 200 SSRs, and we used 6 samples of Iron-Heart *C. lanceolata* for the primer screening. We used Chinese fir plants from six different origins to verify the polymorphism of the primers. We performed PCR amplifications in a 20 µl reaction volume containing 4 µl of template DNA, 10 µl of 1 × Tap PCR Mix (Tiangen) DNA, 1.0 µl of each primer, and 4 µl sterile distilled water. We performed the amplification on an Applied Biosystems 9700 thermocycler with the following touchdown protocol: 95°C for 5 min, followed by 35 cycles of 94 °C for 30 s, 65–55 °C and 72 °C for 30s, then extension at 60°C for 10 min, and finally storage at 4°C. We separated the PCR products on an 8% polyacrylamide gel. We stained the gels with silver nitrate, as previously described [38]. ~~Sangon in Shanghai completed the primer synthesis and PCR product detection.~~

Please show detailed methodology from DNA extraction itself.

Statistical Analysis

We used GeneMarker 2.20 software [39] to read the genotyping results. We observed the number of alleles (NA), observed heterozygosity (HO), expected heterozygosity (HE), and genetic distance matrix between the samples using GenALEx 6.5 [40], and we then used MEGA software [41] for the cluster analysis using the unweighted pair group method with arithmetic mean (UPGMA) according to the genetic distance. We used the online software ITOL [42] to draw and edit the graphics to beautify the cluster analysis structure diagram.

Results

Transcriptome data assembly and unigene annotation

We obtained a total of 45,326,576 clean reads from the transcriptome data of the Iron-Heart *C. lanceolata* leaves, with an average GC content of 44.94% and a Q30 value of 93.62%, which indicated that the sequencing quality was high and could be used. We obtained 184,918 transcripts by ~~splicing with Trinity,~~ and we obtained 115,501 unigenes with an average length of 654.62 bp.

The lengths of N10–N50 were 4190 bp, 3049 bp, 2351 bp, 1800 bp, and 1258 bp, respectively; in other words, the quality of the data assembly was better (Table 2).

Table 2. Distribution characteristics of Iron-Heart *C. lanceolata transcriptome.*

Item	Number or Length
Raw reads	45,422,614
Clean reads	45,326,576
Transcripts	184,918
Unigenes	115,501
N10	4,190 bp
N20	3,049 bp
N30	2,351 bp
N40	1,800 bp
N50	1,258 bp
Average unigene	654.62 bp
GC content	44.94%
Q30	93.62%

~~We completed the gene function annotation of 115,501 unigenes in five databases.~~ We annotated 6,693 unigenes in all five databases, accounting for 5.79% of the total, and 26,278 unigenes in at least one database, accounting for 22.75% of the total (Figure 1). The number of successful annotations in the GO database was the largest (23,962), accounting for 20.75% of the total, and only 8.7% were successfully annotated in Pathway.

Needs strict English language editing here.

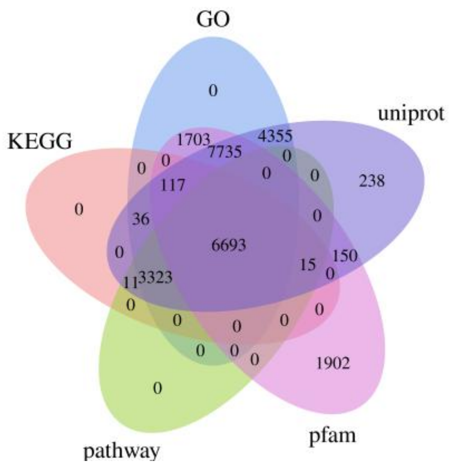

Figure 1. Venn diagram of unigene annotations against Uniprot, Pfam, KEGG, GO, and Pathway databases.

3.2. Gene Ontology (GO) Enrichment and Kyoto Encyclopedia of Genes and Genomes KEGG Pathway Analysis of Unigenes

We performed the GO functional classification of the unigenes using Blast2 GO software [43]. Among them, we annotated 23,963 unigenes in the GO database, which were classified into three main categories (biological processes, cellular components, and molecular functions), as well as 30 subcategories (Figure 1, Figure 2A). All the annotated unigenes had a wide range of functions: metabolic processes and cellular processes under the biological process classification; cells and cellular components under the cellular classification; the binding force (ATP \RNA\zinc ion) and endonuclease in the molecular function category.

To further understand the biological function of Iron-Heart *C. lanceolata*, we compared all the unigenes in the KEGG and Pathway databases. According to the results, 10,196 unigenes (14.33%) were significantly matched and could be placed into five categories: the largest category consisted of BRITE hierarchies, of which three subcategories had a total of 8,001 annotated unigenes, which was followed by the metabolism, genetic information processing, cellular processes, and environmental information processing categories. The KEGG functional analysis provided an important resource for studying the specific processes, pathways, and molecular functions involved in the Iron-Heart *C. lanceolata* transcriptome (Figure 2B).

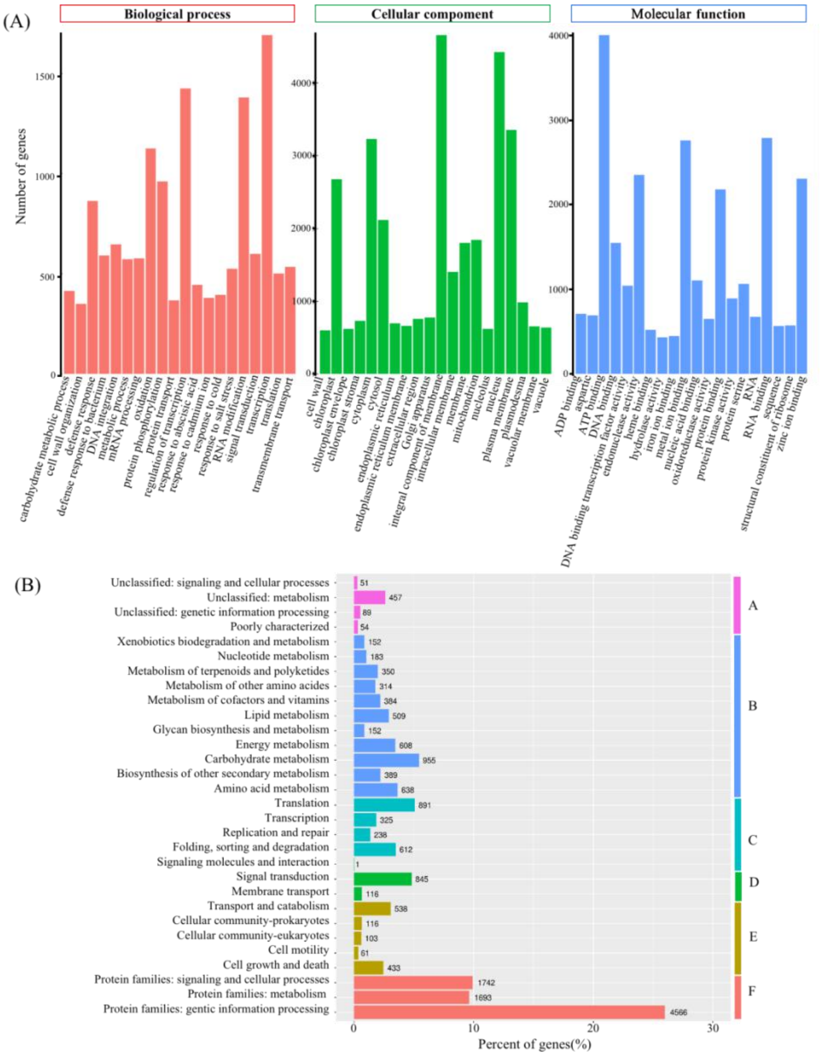

Figure 2. GO annotations of unigenes and clusters of orthologous groups based on KEGG classification: (**A**) not included in Pathway or BRITE; (**B**) metabolism; (**C**) genetic information processing; (**D**) environmental information processing; (**E**) cellular processes; (**F**) BRITE hierarchies.

SSR identification in Iron-Heart *C. lanceolata*

We searched 115,501 unigenes for SSR loci, and we obtained a total of 17,362 SSRs, with a distribution frequency of 4.88%, an average length of 14.28 bp, and an average SSR distribution distance of 4.35/kb. ~~The SSR repeat types were abundant (Table 3).~~ Among them, there were 11,717 single nucleotides, accounting for 67.49% of the total, 2,957 trinucleotides, accounting for 17.03% of the total, and 35 hexanucleotides, which was the minimum number of repeats, accounting for 0.20% of the total. The 17,362 SSRs contained a total of 190 repeat motif types, and the repeat motif types of each nucleotide were 4, 11, 59, 49, 17, and 50, respectively (Table 3).

Among them, the frequency of the AT/TA repeat motif was the highest (960 times), and it was slightly higher than that of the AG/CT motif (873 times). The main repeat motif type of the trinucleotides was AAG/CTT (740 times), with almost twice as many repeats as the AGG/CCT repeat motif type (474 times). The main types and numbers of the TTNR–HXNR repeats were as follows: AAAT/ATTT (35); AAGAG/CTCTT (10); AAGAGG/CCTCTT (20). We ranked the changes in the frequency of the occurrence and distribution density of each repeat type from small to large as follows: pentanucleotides, hexanucleotides, tetranucleotides, dinucleotides, trinucleotides, and mononucleotides. The ranking of the average distance of each repeat type from small to large was as follows: mononucleotides, trinucleotides, dinucleotides, tetranucleotides, hexanucleotides, and pentanucleotides. The overall change rule was that the distribution density increased with the increase in the number of SSR loci, and the change trend of the average distance was the opposite.

Table 3. Distribution of SSR motifs in Iron-Heart *C. lanceolata* transcriptome.

Repeat Motif	SSR Number	Proportion (%)	Frequency (%)	Average Distance (kb)	Distribution Density	Average Length	Repeat Type	Main Repeat Type
MNRs	11717	67.49	10.14	6.45	154.97	12.64	4	A/T
DNRs	2341	13.48	2.03	32.30	30.96	16.70	11	AT/TA (960)
TNRs	2957	17.03	2.56	25.57	39.11	17.27	59	AAG/TTC (740), AGG/CCT (474)
TTNRs	180	1.04	0.16	420.05	2.38	23.87	49	AAAT/ATTT (35)
TTNRs	35	0.20	0.03	2160.26	0.46	25.86	17	AAGAG/CTCTT (10)
HXNRs	132	0.76	0.11	572.80	1.75	34.23	50	AAGAGG/CCTCTT (20)
Total	17362	100.00	15.03	4.35	229.63	14.28	190	

Note: MNRs: mononucleotides; DNRs: dinucleotides; TNRs: trinucleotides; TTNRs: tetranucleotides; RTNR: pentanucleotides; HXNRs: hexanucleotides.

Analysis of Repeat Motif

We also evaluated the frequencies of EST-SSRs with different numbers of tandem repeats, and we present them in Figure 3A. We observed large differences in the repetition numbers of the

SSR repeat types, and the loci formed by the different repetition times were quite different. The numbers of repetitions were from 5 to 84 (Figure 3B). The numbers of repetitions of the single nucleotides were from 10 to 13; the numbers of repetitions of the dinucleotides were from 6 to 13; the numbers of repetitions of the trinucleotides were from 5 to 9, which were relatively large numbers; the numbers of repetitions of the four, five, and six nucleotides were mainly 5 times, which were relatively large numbers. Above all, the SSRs that had from 5 to 10 tandem repeats per locus were the most common types, accounting for 58.96% of the total SSRs, followed by 11–15 tandem repeats (31.34%). The other repetitions accounted for less than 6%. The overall changeable rule was that as the number of repetitions increased, the repetition frequency decreased (Table S1).

We further analyzed the nucleotide repeats for the SSR lengths. The sizes of all the nucleotide repeats were from 10 to 84 bp, and the length of each repeating base was from 10 to 84 bp; from 12 to 74 bp; from 15 to 63 bp; from 20 to 80 bp; from 25 to 55 bp; from 30 to 54 bp, respectively. Only 3.84% of the total number of SSR lengths were greater than or equal to 30 bp, and the remaining 96.16% of the SSR fragments were between 10 and 29 bp in length. Among them, the sizes of the single nucleotides were 10–12 bp, accounting for 48.89% of the total nucleotides, and accounting for 72.44% of the single nucleotides. The sizes of the dinucleotides were about 12 bp, accounting for 6.23% of the total nucleotides, and 46.18% of the dinucleotides. The trinucleotide repeats were mainly 15 bp in size (about 1,751), accounting for 10.09% of the total nucleotides. The main sequence lengths were 20 bp, 25 bp, and 30 bp, respectively, and their proportions in the total nucleotides were all less than 1%.

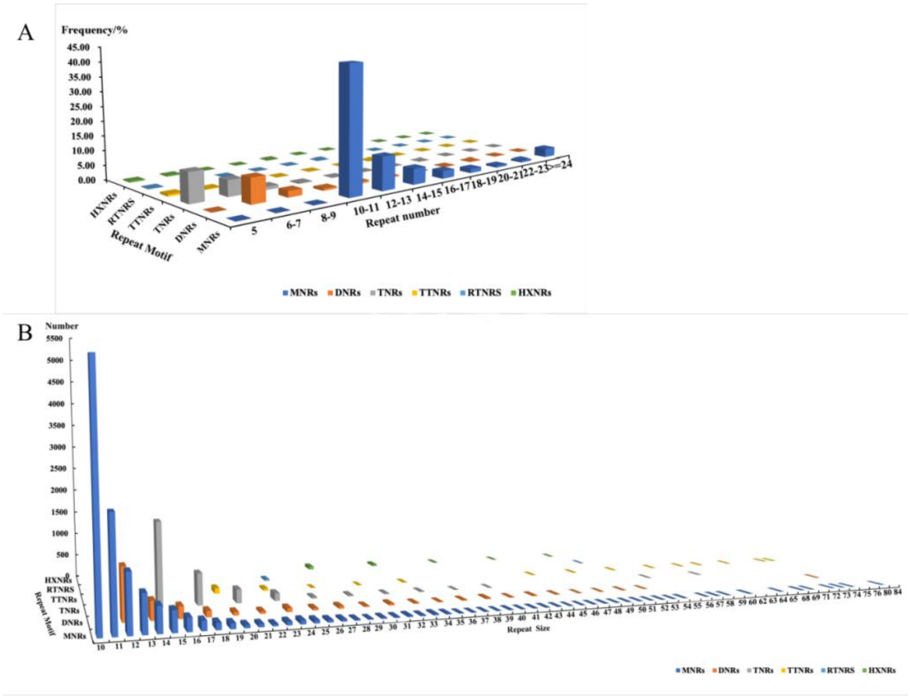

Figure 3. (**A**) Frequencies of SSR repeat types in Iron-Heart *C. lanceolata* transcriptome; (**B**) numbers of SSR repeat types in Iron-Heart *C. lanceolata* transcriptome.

EST-SSR Versatility Detection and Application

To verify the effectiveness and transferability of the primers, we performed PCR amplification and primer screening based on Iron-Heart *C. lanceolata* DNA. We finally selected 15 SSRs to synthesize the fluorescence labeling and detect the polymorphisms in 32 Chinese fir plants of 6 origins, and the amplification rate was only 7.5%. We present the basic information on the 15 SSRs in Table S2. We amplified four capillary electrophoresis templates using IHSSR03 (Figure S1). We identified a total of 51 alleles by 15 pairs of SSR primers. The number of effective alleles was between 1.128 and 2.851, and the average observed heterozygosity and average expected heterozygosity were 0.301 and 0.355, respectively (Table S2). According to the results of the cluster analysis, we could divide the 32 materials into 3 large groups (Figure 4). The genetic distance between Iron-Heart *C. lanceolata* and red-heart Chinese fir was the nearest, which means that their relationship is the closest. In addition, we gathered the samples from Fujian together, and we could use the SSR markers developed from the Iron-Heart *C. lanceolata transcriptome* to effectively distinguish them from the other Chinese fir plants of different origins.

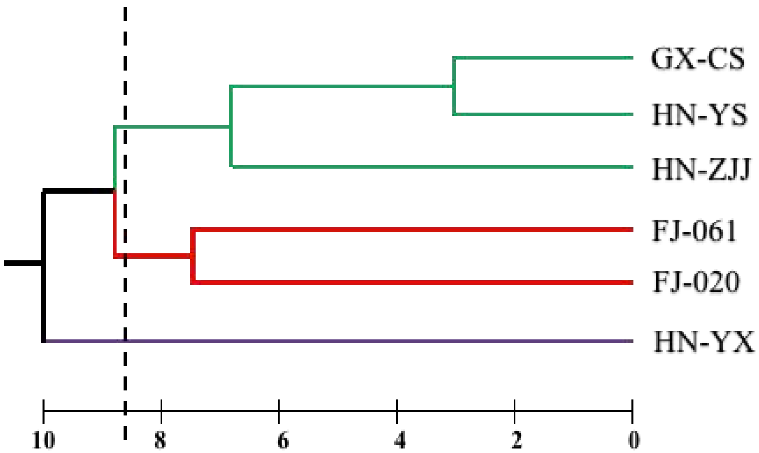

Figure 4. Hierarchical clustering analysis using UPGMA based on 15 EST-SSR markers of 32 samples.

Discussion

Unigene assembly and annotation

Transcriptome sequencing is a high-throughput sequencing technology that researchers use to obtain the transcripts of biological tissues or cells. We can use the coding sequence (CDS) obtained by sequencing to study the differential expression, regulation mode, and functional gene mining under different environments, and to develop markers, such as SSRs and SNPs [28,44,45,46]. The EST-SSR markers developed based on transcriptome sequencing may be directly related to functional genes [46,47,48], and they directly reflect the differences in the transcriptome regions without library construction or screening. In this study, we used the Illumina NovaSeq 6000 high-throughput sequencing platform to sequence the transcriptome of the annual leaves of Iron-Heart *C. lanceolata*, and we developed SSR markers based on the analysis of the transcriptome data. ~~We obtained a total of 45,422,614 raw reads, and we finally obtained 45,326,576 clean reads after stringent quality control and data filtering. The Q30 value was 93.62%, more than 80%, which meant that the sequencing quality was accurate and reliable, the N50 was equal to 1258 bp, and the GC content was 44.94%.~~ The effect of the de novo transcriptome assembly was good, which meant that we could use it. We finally obtained 115,501 unigenes based on the transcriptome data. To further study the biological relevance of the unigenes, we completed sequence alignments and gene function annotations using five databases. According to the GO analysis, there were 23,963 unigenes that were successfully matched, which

Please do not repeat results here.

were divided into three categories according to their functions: biological processes, cellular components, and molecular functions, with 20 subcategories. In the comparison and analysis results of the KEGG and Pathway databases, a total of 10,196 unigenes were annotated, covering BRITE hierarchies, cellular processes, environmental information processing, genetic information processing, and metabolism.

Marker Discovery

EST-SSR markers are one of the tools that we can use to conduct analyses on the genetic structures and fine spatial genetic structures of species, create fingerprint maps, and identify the male parents of offspring [9,10]. At present, there are no SSR markers for Iron-Heart *C. lanceolata*, which greatly limits the work of molecular-assisted breeding and ex situ conservation. In this study, we discovered a total of 17,362 SSR markers and 5,645 polynucleotide repeat motifs in the 115,501 unigenes of Iron-Heart *C. lanceolata*. The SSR frequency was 4.88%, which is comparable to that of Korean pine [21], Masson pine [33], Taxus cuspidata [49], Pinus elliottii Engelm [50], and red-heart Chinese fir [51]; however, it was lower than the SSR distribution frequencies of peony [52], ginger [53], mung bean [54], and other crops. The database size, SSR site search software and conditions, and different organizations all have impacts on the frequency of the SSR distribution [55]. Based on the Iron-Heart *C. lanceolata* transcriptome data, we obtained the SSR markers of six types of repeat motifs. The repeat ratios of the nucleotides were quite different, and the proportion of single nucleotides was the highest at 67.49%, which is consistent with the results of Chen Xingbin [51]. The differences in the distribution of the SSR sequences in different species may be related to the size of the genome differences in the species. Differences in the genome size and base ratio cause substantial differences in the distributions of the dominant SSR sequences.

Pl. check Wen et al. 2015, Scientia Silvae Sinicae; Zeng et al. 2023, Forests; Lin et al. 2020, Scientific Reports

Italics missing

But it can be also due to Illumina sequencing (homopolymer formation)

The AT/TA motif was the main dominant dinucleotide repeat motif of Iron-Heart *C. lanceolata*, accounting for 5.53% of the total number of SSRs, while the CG/CG motif appeared only once in this study. The AAG/CCT and AGG/CTT motifs were the main repeating motifs of the trinucleotides, while the ATC/GAT motif also appeared at a higher frequency in this study, accounting for 2.49% of the total SSRs, which was similar to the results of a study on the precious material *Michelia macclurei* [56]. We also found 118 CCGs/CCGs in the EST sequence of

Iron-Heart *C. lanceolate* (Table S2). This phenomenon was substantially more pronounced in the monocotyledonous plants than in the dicotyledonous plants, and its content was higher than that in ramie [57]. Their existence may be related to specific functions, such as stress resistance, cold resistance, or signaling and transduction; however, we require further related research for verification.

Causes of SSR polymorphisms

The different repeating types and different repeating times are the two main reasons for the polymorphism of SSR markers, which results in high sequence polymorphism. The number of SSR alleles increases with the number of core sequence repeats, which indicates a positive correlation [58]. The abundance of EST-SSRs in this study decreased with the increase in the number of repeat types, and in the same repeat nucleotide repeats, the number of SSR occurrences decreased with the increase in the number of repeats. The variation in SSRs of different lengths facilitates the development of SSR markers with high polymorphism. In the development of the SSR primers, we excluded single nucleotides because they were prone to mismatches, and the remaining SSR repeats were mainly concentrated from 5 to 13 times, and even as high as 25 times. In terms of the length of the SSR fragments, in this study, 88.96% of the SSR fragments were less than 20 bp, and 25.64% of the sequences were 2–6 nucleotide repeats.

Transferability of SSR markers and relationship identification of Chinese fir plants of six different origins

To verify the generality and polymorphism of the SSRs developed based on the Iron-Heart *C. lanceolata* transcriptome, we randomly selected 200 SSR markers, and we identified 15 stable, specific, and polymorphic markers. We developed and analyzed the genetic relationship among the different Chinese fir plants of six origins. The observed heterozygosity and expected heterozygosity of the 15 SSR markers in the 32 samples were 0.301 and 0.335, respectively, which are equal to the results of Xuyang's study on Chinese fir plants of 12 origins [59], and far lower than those in Duan Hongjing's study [60]. The results may be related to the number of markers and populations utilized, the population structures, and interpopulation affinities. Based

on the UPGMA analysis of the 15 SSR markers, we divided the samples into three groups. The phylogenies of the red-heart Chinese fir and Iron-Heart *C. lanceolat* were relatively close; however, this finding requires further research. Researchers have found that their wood densities are comparable [2], but the difference between them requires further study.

Conclusion

We successfully annotated 26,278 out of 115,501 unigenes in five databases. Based on these unigenes, we identified a total of 17,362 EST-SSRs, we randomly selected 200 SSR primers, and we screened 15 pairs of polymorphic primers. We conducted the genetic relationship identification on different origins of Chinese fir. According to the clustering results, 15 SSR markers could effectively distinguish between the Chinese fir varieties of different origins, and the relative relationship between red-heart Chinese fir and Iron-Heart *C. lanceolata* was the closest; however, the difference between them requires further study to comb the phenotyping and molecular means. To the best of our knowledge, although this is not the first time that SSR markers have been developed based on the Chinese fir transcriptome, it is the first attempt to utilize the transcriptome databases and develop a set of EST-SSR markers for Iron-Heart *C. lanceolata.* The research results lay a foundation for analyses on the fine spatial structure, population genetic structure, and molecular-marker-assisted breeding of Iron-Heart *C. lanceolata.*

Clarity missing

Author Contributions

Conceptualization: N.Z., G.H., X.C..
Investigation: G.X., Y.G., S.L..
Data curation: C.X., S.L..
Formal analysis: S.L., G.X..
Writing—original draft preparation: S.L., X.C..
Writing—review and editing: S.L., N.Z., X.C..
Visualization: N.Z..
Funding acquisition: N.Z., X.C..

Funding

This work was supported by the technology innovation project from Hu Nan Forestry Bureau(XLK201921) and Study and Demonstration on Cultivation Techniques of *Cunninghamia lanceolata* Mixed Forest(National key R&D project No:2021YFD2201303-02)

Data Availability Statement

The data presented in this study are available on request from the corresponding author.

Conflicts of Interest

The authors declare no conflict of interest.

References

1. Zhang X, Peng JY, Shi JT, Xu QQ, Xu ZK, Huang F.Wood anatomical characteristics and Physical-mechanical properties of dark-brown heart *Cunningham lanceolata* from Hunnan.Journal of Southwest Forestry University(Natural Sciences). 2021, 41, 155-160.https://kns.cnki.net/kcms/detail/53.1218.S.20200731.1007.006.html
2. You R, Zhu NH, Deng XW, Wang J, Liu F. Variation in wood physical properties and effects of climate for different geographic sources of Chinese fir in subtropical area of China. Scientific reports, 2021, 11(1): 1-11.https://doi.org/10.1038/s41598-021-83500-w
3. Zhu NH, Yang X, Han Z, Han ZQ, C X. Research Progress on Iron-Heart *Cunninghamia lanceolate*. Conifers-Recent Advances, 2021.https://doi.org/10.5772/intechopen.101286
4. Huang RC, Zhu NH, Yang J, Yang XW.Study on cone and seed qualities among different families of black-heart wood Chinese fir. Hunan Forestry Science & Technology.2021,48,40-47.
5. Tautz D. Hypervariability of simple sequences as a general source for polymorphic DNA markers. Nucleic acids research, 1989, 17(16): 6463-6471.https://doi.org/10.1093/nar/17.16.6463
6. Kashi Y, King DG. Simple sequence repeats as advantageous mutators in evolution. TRENDS in Genetics, 2006, 22(5): 253-259.https://doi.org/10.1016/j.tig.2006.03.005
7. Han ZQ, Gao P, Geng XN, Du K, Kang XY. Identification of the male parent of supe

rior half-sib *Populus tomentosa* individuals based on SSR markers. Molecular breeding, 2017, 37(12): 1-12.https://doi.org/10.1007/s11032-017-0754-1

8. Rudolf-Pilih K, Petkovšek M, Jakše J, Štajner N, Murovec J, Bohanec B. Proposal of a new hybrid breeding method based on genotyping, inter-pollination, phenotyping and p aternity testing of selected elite F1 hybrids. Frontiers in plant science, 2019, 10: 1111. 2019,10,1111.https://doi.org/10.3389/fpls.2019.01111

9. Ali A, Pan YB, Wang QN, Wang JD, Chen JL, Gao SJ. Genetic diversity and populati on structure analysis of Saccharum and Erianthus genera using microsatellite (SSR) mar kers. Scientific reports, 2019, 9(1): 1-10.2019, 9,1-10.https://doi.org/10.1038/s41598-018-36630-7

10. Wu QC, Zang FQ, Ma Y, Zheng YQ, Zang DK. Analysis of genetic diversity and po pulation structure in endangered *Populus wulianensis* based on 18 newly developed ES T-SSR markers. Global Ecology and Conservation, 2020, 24: e01329.https://doi.org/10.1 016/j.gecco.2020.e01329

11. Zietkiewicz E, Rafalski A, Labuda D. Genome fingerprinting by simple sequence repea t (SSR)-anchored polymerase chain reaction amplification. Genomics, 1994, 20(2): 176-183.https://doi.org/10.1006/geno.1994.1151

12. Rauscher G, Simko I. Development of genomic SSR markers for fingerprinting lettuce (*Lactuca sativaL.*) cultivars and mapping genes. BMC plant biology, 2013, 13(1): 1-11. https://doi.org/10.1186/1471-2229-13-11

13. Liu SR, Liu HW, Wu AL, Hou Y, An YL, Wei CL. Construction of fingerprinting fo r tea plant (*Camellia sinensis*) accessions using new genomic SSR markers. Molecular Breeding, 2017, 37(8): 1-14.https://doi.org/10.1007/s11032-017-0692-y

14. Jun TH, Van KJ, Kim MY, Lee SH, Walker DR. Association analysis using SSR mar kers to find QTL for seed protein content in soybean. Euphytica, 2008, 162(2): 179-19 1.https://doi.org/10.1007/s10681-007-9491-6

15. Tabkhkar N, Rabiei B, Samizadeh Lahiji H, Hosseini Chaleshtori M. Genetic variation and association analysis of the SSR markers linked to the major drought-yield QTLs of rice. Biochemical genetics, 2018, 56(4): 356-374.https://doi.org/10.1007/s10528-018-9849-6

16. Folta KM, Staton M, Stewart PJ, Jung S, Bies DH, Jesdurai C, et al. Expressed sequence tags (ESTs) and simple sequence repeat (SSR) markers from octoploid strawberry (*Fragaria× ananassa*). BMC Plant Biology, 2005, 5(1): 1-11.https://doi.org/10.1186/1471-2229-5-12
17. Parthiban S, Govindaraj P, Senthilkumar S. Comparison of relative efficiency of genomic SSR and EST-SSR markers in estimating genetic diversity in sugarcane. 3 Biotech, 2018, 8(3): 1-12.https://doi.org/10.1007/s13205-018-1172-8
18. Ditta A, Zhou ZL, Cai XY, Wang XX, Okubazghi KW, Shehzad M, et al. Assessment of genetic diversity, population structure, and evolutionary relationship of uncharacterized genes in a novel germplasm collection of diploid and allotetraploid Gossypium accessions using EST and genomic SSR markers. International journal of molecular sciences, 2018, 19(8): 2401.https://doi.org/10.3390/ijms19082401
19. Li D, Deng Z, Qin B, Liu XH, Men ZH. De novo assembly and characterization of bark transcriptome using Illumina sequencing and development of EST-SSR markers in rubber tree (*Hevea brasiliensis Muell. Arg.*). BMC genomics, 2012, 13(1): 1-14.https://doi.org/10.1186/1471-2164-13-192
20. Xiang XY, Zhang ZX, Wang ZG, Zhang XP, Wu GL. Transcriptome sequencing and development of EST-SSR markers in *Pinus dabeshanensis*, an endangered conifer endemic to China. Molecular Breeding, 2015, 35(8): 1-10.https://doi.org/10.1007/s11032-015-0351-0
21. Du J, Zhang Z, Zhang HG, Junhong T. EST–SSR marker development and transcriptome sequencing analysis of different tissues of Korean pine (*Pinus koraiensis Sieb. et Zucc.*). Biotechnology & Biotechnological Equipment, 2017, 31(4): 679-689.https://doi.org/10.1080/13102818.2017.1331755
22. Wen MF, Wang HY, Xia ZQ, Zou ML, Lu C, Wang WQ. Developmenrt of EST-SSR and genomic-SSR markers to assess genetic diversity in Jatropha Curcas L. BMC research notes, 2010, 3(1): 1-8.https://doi.org/10.1186/1756-0500-3-42
23. Nandha PS, Singh J. Comparative assessment of genetic diversity between wild and cultivated barley using g SSR and EST - SSR markers. Plant breeding, 2014, 133(1): 28-35.https://doi.org/10.1111/pbr.12118

24. McCouch SR, Teytelman L, Xu YB, Lobos KB, Clare K, Walton M, et al. Development and mapping of 2240 new SSR markers for rice (*Oryza sativa L.*). DNA research, 2002, 9(6): 199-207.https://doi.org/10.1093/dnares/9.6.199
25. Song QJ, Shi JR, Singh S, Fickus EW, Costa JM, Lewis J, et al. Development and mapping of microsatellite (SSR) markers in wheat. Theoretical and applied genetics, 2005, 110(3): 550-560.https://doi.org/10.1007/s00122-004-1871-x
26. Liang XY, Bai TD, Wang JZ, Jiang WX. Genome survey and development of 13 SSR markers in *Eucalyptus cloeziana* by NGS. Journal of Genetics, 2022, 101(2): 1-11.https://doi.org/10.1007/s12041-022-01382-x
27. Fluch, S.; Burg, A.; Kopecky, D.; Homolka, A.; Spiess, N.; Vendramin, G.G. Characterization of variable EST SSR markers for Norway spruce (*Picea abies L.*). BMC Research notes. 2011,4,1-6. https://doi.org/10.1186/1756-0500-4-401
28. Wang Z, Gerstein M, Snyder M. RNA-Seq: a revolutionary tool for transcriptomics. Nature reviews genetics, 2009, 10(1): 57-63.https://doi.org/10.1038/nrg2484
29. Tariq MA, Kim HJ, Jejelowo O, Pourmand N. Whole-transcriptome RNAseq analysis from minute amount of total RNA. Nucleic acids research, 2011, 39(18): e120-e120.https://doi.org/10.1093/nar/gkr547
30. Kaur S, Cogan NOI, Pembleton LW, Shinozuka M, Savin KW, Materne M, et al. Transcriptome sequencing of lentil based on second-generation technology permits large-scale unigene assembly and SSR marker discovery. BMC genomics, 2011, 12(1): 1-11.https://doi.org/10.1186/1471-2164-12-265
31. Wu J, Cai CF, Cheng FY, Cui HL, Zhou H. Characterisation and development of EST-SSR markers in tree peony using transcriptome sequences. Molecular Breeding, 2014, 34(4): 1853-1866.https://doi.org/10.1007/s11032-014-0144-x
32. Zhang Z, Zhang HG, Mo C, Zhang L. Transcriptome sequencing analysis and development of EST-SSR markers for *Pinus koraiensis*. Scientia Silvae Sinicae, 2015, 51(8): 114-120.
33. Mei LN, Fan FH, Cui BW, Wen XP. Development of SSR molecular markers based on transcriptome sequences and germplasm identification in masson pine (*Pinus massoniana*). Journal of Agricultural Biotechnology.2017,25,991-1002.(in Chinese)

34. Wen Y, Ueno S, Han W, Tsumura Y. Development and characterization of 28 polymorphic EST-SSR markers for *Cunninghamia lanceolata* (Taxodiaceae) based on transcriptome sequences. Silvae Genetica, 2013, 62(1-6): 137-141. https://doi.org/10.1515/sg-2013-0018

35. Yang XW. Construction of Parent Population for Breeding Programme in the Iron-heart Chinese fir. Central South University of Forestry and Technology,2021.DOI:10.27662/d.cnki.gznlc.2021.000470.

36. Grabherr MG, Haas BJ, Yassour M, Levin JZ, Thompson DA, Amit I, et al. Full-length transcriptome assembly from RNA-Seq data without a reference genome. Nature biotechnology, 2011, 29(7): 644-652.https://doi.org/10.1038/nbt.1883

37. Davidson NM, Oshlack A. Corset: enabling differential gene expression analysis for de novoassembled transcriptomes. Genome biology, 2014, 15(7): 1-14.https://doi.org/10.1186/s13059-014-0410-6

38. Bassam BJ, Caetano-Anollés G, Gresshoff PM. Fast and sensitive silver staining of DNA in polyacrylamide gels. Analytical biochemistry, 1991, 196(1): 80-83.https://doi.org/10.1016/0003-2697(91)90120-I

39. Holland MM, Parson W. GeneMarker® HID: A reliable software tool for the analysis of forensic STR data. Journal of forensic sciences, 2011, 56(1): 29-35.https://doi.org/10.1111/j.1556-4029.2010.01565.x

40. Smouse R, Peakall R. GenAlEx 6.5: genetic analysis in Excel. Population genetic software for teaching and research—an update. Bioinformatics. 2012,28,2537-2539.https://doi.org/10.1093/bioinformatics/bts460

41. Tamura K, Dudley J, Nei M, Kumar S. MEGA4: molecular evolutionary genetics analysis (MEGA) software version 4.0. Molecular biology and evolution, 2007, 24(8): 1596-1599.https://doi.org/10.1093/molbev/msm092

42. Letunic I, Bork P. Interactive Tree Of Life (iTOL) v4: recent updates and new developments. Nucleic acids research, 2019, 47(W1): W256-W259.https://doi.org/10.1093/nar/gkz239

43. Conesa A, Götz S. Blast2GO: a comprehensive suite for functional analysis in plant genomics. International journal of plant genomics, 2008, 2008.https://doi.org/10.1155/200

8/619832

44. Marguerat S, Bähler J. RNA-seq: from technology to biology. Cellular and molecular l ife sciences, 2010, 67(4): 569-579.https://doi.org/10.1007/s00018-009-0180-6

45. Haas BJ, Zody MC. Advancing RNA-seq analysis. Nature biotechnology, 2010, 28(5): 421-423.https://doi.org/10.1038/nbt0510-421

46. Wang D, Yang C, Dong L, Zhu JC, Wang JP, Zhang SF. Comparative transcriptome analyses of drought-resistant and-susceptible *Brassica napus L.* and development of ES T-SSR markers by RNA-Seq. Journal of Plant Biology, 2015, 58(4): 259-269.https://do i.org/10.1007/s12374-015-0113-x

47. Cai K, Zhu LF, Zhang KK, Li L, Zhao ZY, Zeng W, et al. Development and charact erization of EST-SSR markers from RNA-Seq data in *Phyllostachys violascens*. Fronti ers in plant science, 2019, 10: 50.https://doi.org/10.3389/fpls.2019.00

48. Han SM, Wu ZJ, Jin Y, Yang WN, Shi HZ. RNA-Seq analysis for transcriptome asse mbly, gene identification, and SSR mining in ginkgo (*Ginkgo biloba L.*). Tree Genetic s & Genomes, 2015, 11(3): 1-10.https://doi.org/10.1007/s11295-015-0868-8

49. Ueno S, Wen YF, Tsumura Y. Development of EST-SSR markers for *Taxus cuspidata* from publicly available transcriptome sequences. Biochemical Systematics and Ecolog y, 2015, 63: 20-26.https://doi.org/10.1016/j.bse.2015.09.016

50. Yi M, Zhang L, Lei L, Chen ZS, Sun SW, Lan M. Analysis of SSR information in t ranscriptome and development of EST-SSR molecular markers in *Pinus elliottii Engel m*.Journal of Nanjing Forestry University (Natural Sciences Edition).2020,44,75-83.DOI: 10. 3969 /j. issn. 1000－2006. 201907017(in Chinese)

51. Chen XB, He L, Xiao FM, Lou YF, Xu HN, Sun SW. Development and application of EST-SSR markers in chenshan red-heart Chinese fir based on transcriptome sequen cing. Journal of Central South University of Forestry & Technology. 2020,40,120-127. Doi:10.14067/j.cnki.1673-923x.2020.08.015(in Chinese)

52. Wu J, Cai CF, Cheng FY, Cui HN, Zhou H. Characterisation and development of ES T-SSR markers in tree peony using transcriptome sequences. Molecular Breeding, 201 4, 34(4): 1853-1866.https://doi.org/10.1007/s11032-014-0144-x

53. Vidya V, Prasath D, Snigdha M, Gobu R, Sona C, Maiti CS. Development of EST-S

SR markers based on transcriptome and its validation in ginger (*Zingiber officinale R osc.*). Plos one, 2021, 16(10): e0259146.https://doi.org/10.1371/journal.pone.0259146

54. Chen HL, Wang LX, Wang SH, Liu CJ, Blair MW, Cheng XZ. Transcriptome sequencing of mung bean (*Vigna radiate L.*) genes and the identification of EST-SSR markers. PloS one, 2015, 10(4): e0120273.https://doi.org/10.1371/journal.pone.0120273
55. Varshney RK, Sigmund R, Börner A, Korzun V, Stein N, Sorrels ME, et al. Interspecific transferability and comparative mapping of barley EST-SSR markers in wheat, rye and rice. Plant Science, 2005, 168(1): 195-202.https://doi.org/10.1016/j.plantsci.2004.08.001
56. Li QY, Zhong CL, Jiang QB, Zhang Y, Chen Y, Wei YC, et al. Characteristic analysis of microsatellites in the transcriptome of *Michelia macclurei* of rare tree species[J]. Genomics and Applied Biology, 2019, 38(4): 1674-1682.DOI: 10.13417/j.gab.038.001674
57. Xu YJ, Ma YS, Dong JY, Liang B, Zhang Y, Wang YM, et al. SSR Sequence Analysis and EST-SSR Marker Development Based on Ramie Transcriptome Sequencing.Molecular Plant Breeding. 2020,18,4368-4376. DOI: 10.13271 j.mpb.018.004368
58. Xing W, Liao JY, Cai MY, Xia QF, Liu Y, Zeng W, et al. De novo assembly of transcriptome from *Rhododendron latoucheae Franch.* using Illumina sequencing and development of new EST-SSR markers for genetic diversity analysis in Rhododendron. Tree Genetics & Genomes, 2017, 13(3): 1-14.https://doi.org/10.1007/s11295-017-1135-y
59. Xu Y, Chen JH, Li Y, Hong Z, Wang Y, Zhao YQ, et al. Development of EST-SSR and genomic-SSR in Chinese fir. Journal of Nanjing Forestry University (Natural Sciences Edition), 2014, 38(1): 9-14.doi:10.3969 /j.issn.1000-2006.2014.01.002
60. Duan HJ, Hu RY, Wu B, Chen DX, Huang KY, Dai J, et al. Genetic characterization of red-colored heartwood genotypes of Chinese fir using simple sequence repeat (SSR) markers. Genetics and Molecular Research, 2015, 14(4): 18552-18561.https://doi.org/10.4238/2015.December.28.2

Click here to access/download
Supporting Information
Supplementary materials.pdf
