# Supplementary material for: De novo assembly of Iron-Heart Cunninghamia lanceolata transcriptome and EST-SSR marker development for genetic diversity analysis
Source: PLoS One. 2023 Nov 2;18(11):e0293245. doi: 10.1371/journal.pone.0293245 (PMC10621985; doi:10.1371/journal.pone.0293245)
Supplement: Attachment — Submitted filename: Response to Reviewers.pdf [file pone.0293245.s008.pdf]

Response to Reviewers

Dear PH.D Pramod Prasad,

Thank you for offering us once more an opportunity to resubmit a revised manuscript. Here, we submit the revised manuscript entitled “De novo assembly of Iron-Heart *Cunninghamia lanceolata* transcriptome and EST-SSR marker development for genetic diversity analysis” (ID: PONE-D-22-35149) to PLOS ONE.

We appreciate your letter and the reviewers’ comments concerning our manuscript. These comments are all valuable and very helpful for revising and improving our paper, as well as the important guiding significance to our researches. We have studied comments carefully and have made correction which we hope meet with approval. Revised portion are marked by using 'track changes' in the paper.
Detailed responses to associate editor and the two reviewers´ comments are provided in the next sections.

Therefore, I would be greatly appreciated for that you can speed up the review process.We hope you find the improvements to the manuscript satisfactory. Please feel free to contact us with any questions and we are looking forward to your response.

Thank you and best regards.

Yours sincerely,

Can Xiao

E-mail: 17916370@qq.com

Responds to the associate editor's comments:

Responds to the reviewer's comments:

Reviewer #1:

Point 1: whole languages were very poor

Response: We have polished the language of the article in accordance with the standards required by SCI papers.

Point 2: In methods section, the writing of genetic diversity index was nonstandard.

Response: Thank you very much for your valuable comments, we have modified the genetic diversity index according to the standard format in line 139-141 of Manuscript.

Point 3: Within Figure 4, the abbreviation should be noted in the title.

Response: The abbreviations within Figure 4 have presented in Table1 in the Materials and methods section, maybe we did not describe them clearly in the article, now we describe them again in the line 94 of the Manuscript.

Point 4: Discussion section, there were many sentences repeat with the results. Thus, "Unigene assembly and annotation" and "Marker discovery" could be merged.

Response: Thank you very much for your valuable comments, we have revised the language of the article.

Point 5: In "Transferability of SSR markers ……", the genetic diversity level of Iron-Heart *C. lanceolate* far lower than those in Duan Hongjing's study…., "The phylogenies of the red-heart Chinese fir and *Iron-Heart C. lanceolate* were relatively close….." These should be much related the limited samples that make the rationality insufficient

Response: Thank you very much for your valuable suggestions, we have revised the discussion section. What we wanted to express is the relationship of the Iron-Heart *C. lanceolate* and red-heart Chinese were relatively close based on the 15 SSR markers constructed in this study. Of course, this is also related to our limited samples, but we could learn from the UPGMA that these 15 SSR markers can effectively distinguish

Chinese fir from different provenances. Later, we will consider collecting as many samples as possible to carry out further research using the markers of this study.

Responds to the reviewer's comments:

Reviewer #2:

Point 1: English editing of the revision is a must. At most places, clarity is missing and there are insincerities in writing.

Response: First of all, I would like to thank the expert for your valuable opinions, which made us aware of the importance of writing skills and language in SCI papers. We have further organized the language of the paper in accordance with the requirements of SCI paper standards.

Point 2: Title- please edit: De novo assembly of Iron-Heart *Cunninghamia lanceolata* transcriptome and EST-SSR marker development for genetic diversity analysis

Response: The title of the paper suggested by the expert is very consistent with what we want to express. Compared with the previous title, it is more concise and precise.

Point 3: Abstract is data heavy and long. Just discuss brief results for a summative understanding.

Response: In our previous abstract, we tried to express all the meaning of the article, but the content was too redundant. Based on your suggestion we have re-edited the summary.

Point 4: Mononucleotide repeats should not be considered at all. Please focus from di- to hexa-nucleotide repeats.

Response: We have edited the article and focus from di- to hexa-nucleotide repeats.

Point 5: Please include RepeatMasker based repeat analysis (additional data).

Response: Your suggestion is very helpful to us. We know that supplementing this analysis will further prove the reliability of our research. The SSR of Iron-Heart *Cunninghamia lanceolata* identified in this paper can already distinguish the relationship between different species, which has achieved our goal. We are doing the whole work you mentioned, and we will continue to add it in future work.

Point 6: Please include COG-based annotation results.

Response: Thanks to experts for valuable opinions. The COGs were constructed by applying the criterion of consistency of genome-specific best hits to the results of an exhaustive comparison of all protein sequences from these genomes. The database comprises 2091 COGs that include 56–83% of the gene products from each of the complete bacterial and archaeal genomes and ~35% of those from the yeast Saccharomyces cerevisiae genome. The COG database is accompanied by the COGNITOR program that is used to fit new proteins into the COGs and can be applied to functional and phylogenetic annotation of newly sequenced genomes.It is very suitable for this study. In the revised manuscript we added COG-based annotation and redrawn the Venn diagram.

Point 7: Why PAGE and silver nitrate solution was used for determination of PCR products? Why not agarose-based determination?

Response: PAGE and silver nitrate solution have higher resolution, but agarose-based determination has lower resolution. In this study, several SSR primers were of similar size. It is difficult to distinguish polymorphic primers if agarose-based determination is used; In addition, we use PAGE in this study to facilitate our follow-up research.

Point 8: Whether the unigenes obtained were all 'coding for ORFs'?

Response: No

Point 9: How did you differentiate between KEGG and pathway analysis

Response: KEGG is a database resource for understanding high-level functions and utilities of the biological system, such as the cell, the organism and the ecosystem, from molecular-level information, especially large-scale molecular datasets generated by genome sequencing and other high-throughput experimental technologies. And The KEGG pathway is one of KEGG, including molecular interactions and relationship networks related to metabolism, regulation, pathways, biochemistry, diseases, drugs, etc. In other words, the coverage of KEGG is wider than that of KEGG PATHWAY. Therefore, we removed the pathway annotation and added the COG annotation in our revised manuscript.

Point 10: Please present the pfam annotations as a Table (1902 annotations were

unique)

Response: Based on your suggestion, we re-compared the original data with 5 databases such as KEGG, COG and so on, we found that 718 are unique in pfam, and now we presented the unique comparisons of pfam and COG in table in Supplementary Materials.

Point 11: Please provide figure 3A and B as a table and present the motif types as a figure.

Response: Thank you very much for your suggestion, we thought the figure looked more intuitive, but it did ignore clarity. Therefore, according to your suggestion, we have made a revision, and we present the motif types in the form of a figure, in line 207 and of the revised manuscript and summary of EST-SSRs identified in Iron-Heart *C. lanceolata* transcriptome were presented as a table in line 239 of the revised manuscript.

Point 12: Discussion is weak. Please discuss your data with previous findings.

Response: Thank you very much for your valuable opinion. In the revision of revised manuscript, we compared the results of this article with the previous research as much as possible. Iron-Heart *C. lanceolata* is a new native variety of Chinese fir, So far, there are relatively few studies on it.

Point 13: BioProject?

Response: PRJCA016964
