# Supplementary material for: De novo assembly of Iron-Heart Cunninghamia lanceolata transcriptome and EST-SSR marker development for genetic diversity analysis
Source: PLoS One. 2023 Nov 2;18(11):e0293245. doi: 10.1371/journal.pone.0293245 (PMC10621985; doi:10.1371/journal.pone.0293245)
Supplement: Attachment — Submitted filename: Response to Reviewers.docx [file pone.0293245.s009.docx]

Response to Reviewers

Dear PH.D Pramod Prasad,

Thank you for offering us once more an opportunity to resubmit a revised manuscript. Here, we submit the revised manuscript entitled “De novo assembly of Iron-Heart *Cunninghamia lanceolata* transcriptome and EST-SSR marker development for genetic diversity analysis” (ID: PONE-D-22-35149 ) to PLOS ONE.

We appreciate your letter and the reviewers’ comments concerning our manuscript. These comments are all valuable and very helpful for revising and improving our paper, as well as the important guiding significance to our researches. We have studied comments carefully and have made correction which we hope meet with approval. Revised portion are marked by using 'track changes' in the paper.

Detailed responses to associate editor and the two reviewers´ comments are provided in the next sections.

Therefore, I would be greatly appreciated for that you can speed up the review process.We hope you find the improvements to the manuscript satisfactory. Please feel free to contact us with any questions and we are looking forward to your response.

Thank you and best regards.

Yours sincerely,

Xiao Cai

E-mail: 17916370@qq.com

# Responds to the reviewer’s comments:

## Reviewer #3:

**Point 1:** Line # 125-130: explains the 3 objectives. I feel the third objective is redundant and it forms the part of 2nd objective. This could be modified suitably

**Response:** Indeed, we agree with your suggestion very much. After comprehensive consideration, this study mainly has two goals, and we have already made revisions in the line 77-79 in the Revised Manuscript with Track Changes.

**Point 2:** Line #340 & 465: Word transferability is used to denote the SSR markers amplifications in accessions from different regions. But the term transferability is used for their amplification or applicability across species i.e., cross species amplifications. So, usage of this term could be avoided here.

**Response:** Thank you very much for your valuable comments, we have replaced word “transferability” with word “cross-species transferability” in line 243 and line 346 in the Revised Manuscript with Track Changes.

**Point 3:** Table 4: Generally, the SSRs are classified as class I (>20 bp) & ClassII (12-20 bp) based on size of repeat motifs. Which allows the users to select markers. Usually, Class I are more polymorphic. Please add few lines in the results and discussion based on this classification

**Response:**The characteristically short lengths of SSRs may have functional implications with respect to their evolution or the genes involved in plant physiology and development. We also read the article on the classification of grades according to the length of the SSR, and we made the corresponding result analysis and discussion in our Revised Manuscript with Track Changes in the line 237-240 and line 339-345.
